# Association of MMP2 and MMP9 gene polymorphisms with nonsyndromic cleft lip/palate in an Iranian population

**DOI:** 10.34172/joddd.2023.40640

**Published:** 2023-11-11

**Authors:** Fatemeh Zahedipour, Hamid Reza Khorram Khorshid, Emran Esmaeilzadeh, Koorosh Kamali, Asghar Ebadifar

**Affiliations:** ^1^Department of Orthodontics, Shahid Beheshti University of Medical Sciences, Tehran, Iran; ^2^Genetic Research Center, University of Social Welfare and Rehabilitation Sciences, Tehran, Iran; ^3^Fetal Health Research Center, Hope Generation Foundation, Tehran, Iran; ^4^Department of Public Health, School of Public Health, Zanjan University of Medical Sciences, Zanjan, Iran; ^5^Dentofacial Deformities Research Center, Research Institute of Dental Sciences, Department of Orthodontics, Shahid Beheshti University of Medical Sciences, Tehran, Iran

**Keywords:** Cleft lip/palate, MMP2, MMP9, Nonsyndromic, Polymorphism

## Abstract

**Background.:**

Cleft lip/palate (CL/P) is a prevalent congenital disorder. Matrix metalloproteinases (MMPs) play a role in palatogenesis and have been proposed to be associated with nonsyndromic CL/P development. This study aimed to examine the association of MMP2 (rs243866) and MMP9 (rs3918242) gene polymorphism with nonsyndromic CL/P in an Iranian population.

**Methods.:**

Blood samples were collected from 120 nonsyndromic CL/P patients and 140 healthy newborns in this case-control study. DNA extraction was performed by the salting-out method, and the samples underwent polymerase chain reaction (PCR) and restriction fragment length polymorphism (RFLP), using Pag and SphI enzymes, for genotyping MMP2 and MMP9 gene polymorphisms. Statistical analysis was performed with SPSS 11.5. Univariate and multivariate logistic regression models were used to calculate the odds ratios and 95% confidence intervals (CIs). The level of statistical significance was set at *P*<0.05.

**Results.:**

No significant association was found between MMP2 gene polymorphism and nonsyndromic CL/P. However, the MMP9 gene polymorphism had a significant association with nonsyndromic CL/P, with a higher prevalence of the T allele and TT genotype in the case group than the control group.

**Conclusion.:**

This study indicated a potential link between MMP9 gene polymorphism and nonsyndromic CL/P in an Iranian population. Future investigations with greater sample diversity and larger sample sizes are required to obtain more comprehensive and robust evidence. In-depth analyses and studies involving different ethnic groups can further enhance our understanding of the genetic underpinnings of CL/P.

## Introduction

 Cleft lip/palate (CL/P) is among the most common congenital defects believed to have a multifactorial etiology. It occurs as a result of complex interactions of genetics and environmental factors.^1^ It has an incidence of 3.73/1000 live births in the Iranian population.^2,3^ Research utilizing whole genome analysis has identified 18 genetic risk loci associated with CL/P.^4^ Detection of candidate genes for nonsyndromic CL/P (without other related defects) would enable the prediction and prevention of this disorder.^5^

 The role of matrix metalloproteinases (MMPs) in the development of CL/P is a topic of interest due to their involvement in the development of craniofacial structures.^6^ Evidence shows that MMPs 2, 3, 7, 9, and 13 are expressed during the fusion of the palate in mice, both temporally and spatially.^7^ Also, MMPs play a role in the formation of the palate, involving proteolytic degradation of the extracellular matrix (ECM), which is necessary for the fusion of the lip and palate. Changes in MMP levels might theoretically result in the inability of fusion, leading to cleft formation in the lip and/or palate^8^ Such observations show that MMPs might be potentially associated with the etiology of CL/P.

 The MMP9 gene, located on chromosome 20q12.2, is recognized for its capacity to degrade type IV collagen, a significant constituent of the ECM, and stimulates cell migration.^9^

 To date, researchers have identified at least 12 potential single nucleotide polymorphisms (SNPs) in the promoter and coding region of the MMP9 gene that may have clinical significance in its expression and function. A polymorphism on the MMP9 gene promoter at position 1562 (C/T) has been found to have a functional impact on transcription. The substitution of C with T leads to the disruption of the binding of a nuclear protein in this region of the MMP9 gene, resulting in increased transcriptional activity.^9^

 The MMP2 gene, located on chromosome 16q13.21, is another MMP implicated in the pathologies affecting the dentin‒pulp complex; it may also contribute to dental abnormalities in individuals with CL/P.^10^ A common SNP in the CCACC box of the MMP2 gene promoter, 1306 C/T, is involved in CL/P in several populations. This SNP changes the amino acid sequence from alanine to valine at position 429 of the protein.^11-13^ The valine allele has been associated with decreased MMP2 activity and increased risk of CL/P in some investigations^14^ Therefore, the role of MMP2 polymorphism in the development of CL/P is currently a topic of debate and uncertainty, necessitating further research in this regard.

 Considering the role of MMP2 and MMP9 in the development of craniofacial structures and dental abnormalities, it is important to investigate whether genetic variations in these genes could contribute to the development of CL/P. Thus, the current study investigated the occurrence of MMP2 and MMP9 gene polymorphisms in individuals with CL/P compared to healthy controls. By identifying any potential association between specific genetic variations and CL/P, this study could provide insights into the genetic factors underlying this common congenital abnormality.

## Methods

###  Participants

 A total of 260 participants were enrolled, including 120 patients with nonsyndromic CL/P and 140 healthy controls. The minimum sample size for the CL/P group was calculated at 120 patients. All the participants were of Iranian descent. Prior to the analysis, the parents or legal guardians of all the participants provided written consent after being fully informed about the study, and blood samples were collected from the newborns for DNA extraction (Ethics code: IR.SBMU.DRC.REC.1401.061).

 The inclusion criteria for the patients in the CL/P group were as follows:

- Nonsyndromic CL/P
- Iranian ethnicity
- Parental consent for blood collection from their newborn

The inclusion criteria for the control group were as follows:

- No family history of CL/P
- Matching the CL/P patients in terms of age, gender, and geographical region
- Absence of any syndrome
- Parental consent for blood collection from their newborn

###  Genotyping

 DNA was extracted from the blood samples by the salting-out method.^15-18^ The quality and quantity of the extracted DNA were evaluated by gel electrophoresis and spectrophotometry^19,20^

 Polymerase chain reaction (PCR) was performed using Taq DNA polymerase (Boiron, USA), 10-mM dNTP mix (Boiron, USA), 50-mM MgCl_2_ solution (Boiron, USA), and primers (Pishgam, Iran) specific to MMP2 and MMP9 genes, which were designed using Primer 3 and Neb Cutter software. The PCR conditions for each gene were optimized, and PCR was performed for all the samples. Amplification of both MMP2 and MMP9 genes was performed using the following PCR conditions: Initial denaturation at 94 °C for 5 minutes, followed by 35 cycles of denaturation at 94 °C for 45 seconds, annealing at 62 °C for 15 seconds, extension at 72°C for 45 seconds, and final extension at 72 °C for 10 minutes. Confirmation of the PCR products was performed by running them on an agarose gel using electrophoresis.

 Restriction fragment length polymorphism (RFLP) was used to determine the genotype of MMP2 and MMP9 genes. The PCR products were digested with specific restriction enzymes (PagI for MMP2 and SphI for MMP9). The digested fragments were analyzed by 2% agarose gel electrophoresis to identify different genotypes associated with the development of CL/P. Table 1 presents the sequences of primers and the sizes of PCR products, along with the sizes of the RFLP fragments.

**Table 1:** Primer sequences and the corresponding product sizes for MMP2 and MMP9 genes, and the sizes of RFLP products after restriction enzyme digestion

**SNPs**	**Primer sequence (5´→ 3´)**	**Product size (bp)**	**RFLP Fragments (bp)**
MMP2 (rs243866G/A)	F:ACCCACCAGACAAGCCTGAAC R: GATGGAGCTGGAGGGGTCAG	228	GG:228bp AA:142bp + 86bp GA:228bp + 142bp + 86bp
MMP9 (rs3918242C/T)	F: TGGTCAACGTAGTGAAACCCCATCT R: CCAGCCCCAATTATCACACTTAT	385	CC:385 bp TT:317 bp + 68 bp CT:385bp + 317bp + 68bp

###  Data analysis

 Statistical analysis was performed with SPSS 11.5. Univariate and multivariate logistic regression models were used to calculate the odds ratios and 95% confidence intervals (CIs). The level of statistical significance was defined at *P* < 0.05.^21^

## Results

 PCR amplification and RFLP-PCR were performed to analyze MMP2 (rs243866G/A) and MMP9 (rs3918242C/T) gene polymorphisms in the case and control groups. The PCR products were separated on 2% agarose gel, and the RFLP patterns were visualized using specific enzymes (PagI for MMP2 and SphI for MMP9). The genotype frequencies in both groups were found to be in the Hardy-Weinberg equilibrium (*P* < 0.001 for both groups). The results showed variations in the frequencies of alleles and distributions of genotypes of the MMP2 polymorphism when the case and control groups were compared. The MMP2 gene polymorphism analysis revealed three genotypes: the GG (wild-type) genotype was observed in 78 patients (65%) in the case group and 89 individuals (63.6%) in the control group. The GA (heterovariant) genotype was detected in 16 patients (13.3%) in the case group and 15 individuals (10.7%) in the control group, and the AA (variant) genotype was found in 26 patients (21.7%) in the case group and 36 individuals (25.7%) in the control group. The prevalence of the A allele, corresponding to the variant genotype, was higher in the control group (31.1%) compared to the case group (28.3%), but this difference was not statistically significant (*P* = 0.519).

 Moreover, regarding the MMP9 polymorphism, the MMP9 gene polymorphism analysis also revealed three genotypes: the CC (wild-type) genotype was observed in 40 patients (33.3%) in the case group and 92 individuals (65.7%) in the control group, the CT (heterovariant) genotype was detected in 24 patients (20%) in the case group and 18 individuals (12.9%) in the control group, and the TT (variant) genotype was found in 56 patients (46.7%) in the case group and 30 individuals (21.4%) in the control group.

 The prevalence of the T allele, corresponding to the variant genotype, was significantly higher in the case group (56.7%) compared to the control group (27.9%) (*P* < 0.001). Table 2 shows the allele and genotype distribution of MMP2 and MMP9 gene polymorphisms in the case and control groups.

**Table 2:** Allele and genotype distribution of MMP2 and MMP9 gene polymorphisms in the case and control groups

**Gene**	**Genotype**	**Case (n=120)**	**Control (n=140)**
MMP2	GG	78 (65.0%)	89 (63.6%)
	GA	16 (13.3%)	15 (10.7%)
	AA	26 (21.7%)	36 (25.7%)
	Allele	G: 122 (51.7%)	G: 193 (68.9%)
		A: 114 (48.3%)	A: 87 (31.1%)
MMP9	CC	40 (33.3%)	92 (65.7%)
	CT	24 (20.0%)	18 (12.9%)
	TT	56 (46.7%)	30 (21.4%)
	Allele	C: 104 (43.3%)	C: 202 (72.1%)
		T: 136 (56.7%)	T: 78 (27.9%

**Figure 1:**
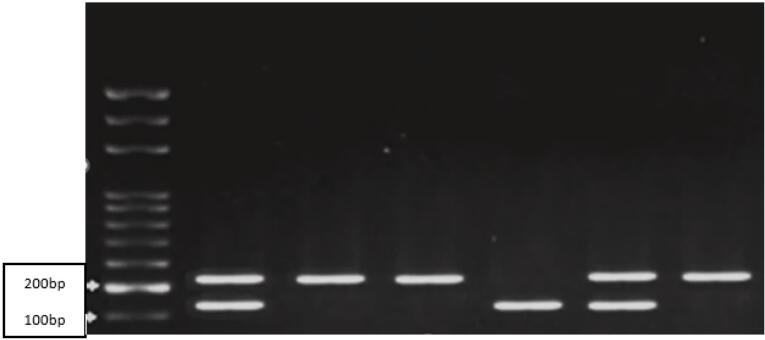
RFLP product analysis using the PagI enzyme for MMP2 gene polymorphism (GG: 228 bp, GA: 142, 228 bp, and AA: 142 bp)

**Figure 2:**
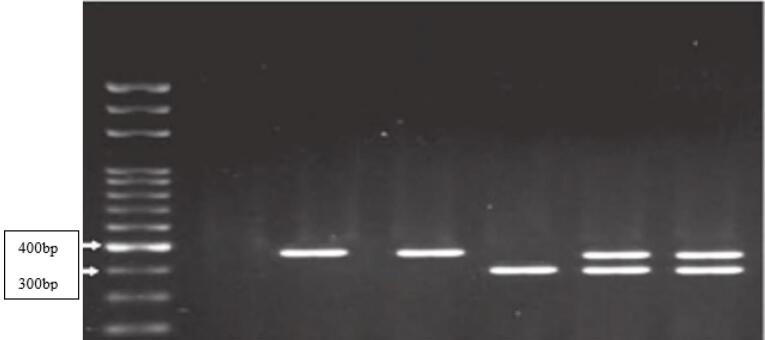
RFLP product analysis using the SphI enzyme for MMP9 gene polymorphism (CC: 385 bp, CT: 317, 385 bp, and TT: 317 bp)

Figures 1 and 2 show the RFLP products generated by the enzymatic digestion for MMP2 and MMP9 gene polymorphisms in a few selected samples of the case group.

## Discussion

 Understanding the complex mechanisms underlying the development of CL/P is essential to unravel the involved genetic and environmental factors. Both biological and human studies provide evidence supporting the candidacy of MMPs and their inhibitors as relevant genes for nonsyndromic CL/P.^22^ Previously, investigations on nonsyndromic orofacial clefts predominantly centered on examining SNPs individually. Therefore, investigating various polymorphisms in these genes and their association with CL/P has become a crucial approach in clinical and basic research. The present study evaluated the rs243866 polymorphism in the MMP2 gene and the rs3918242 polymorphism in the MMP9 gene in nonsyndromic CL/P patients and healthy controls.

 MMP2 and MMP9 are two proteases with a significant role in ECM degradation, including type IV collagen and laminin, which are the main components of the basal membrane.^23^ Upregulation of MMP2, MMP3, MMP9, and MMP13 in the midface has been closely associated with degeneration of the inner epithelium of the palatal shelves during palate fusion.^24^

 Considering the role of MMP2 in cell migration, ECM degradation, and epithelial cell adhesion, it is anticipated that MMP2 would also be expressed during lip development.^8,25,26^ Evidence shows higher levels of MMP2 in the CL/P region of the affected individuals. Therefore, increased MMP2 activity could be considered a destructive factor in the development of nonsyndromic CL/P.^27^ On the other hand, Smane et al^25^ demonstrated that the defective allele A reduces the transcriptional activity and expression of MMP2, leading to lower MMP2 levels. Consistent with their results, the present study found that the defective allele A had a non-significantly higher prevalence in the control group than in the case group. Similarly, comparing the frequencies of GG, GA, and AA genotypes between the patient and control groups did not reveal any statistically significant difference, consistent with the findings of a study by Machado et al^28^ in Brazil, which had a much larger sample size. Thus, this polymorphism does not appear to have a role in the development of nonsyndromic CL/P. However, further investigations on larger and more diverse populations are required with more robust statistical methods to confirm this finding.

 Zalewska-Ziob et al^29^ examined the association of C/T (rs243866) polymorphism or 1575 A/G in various types of cleft lip patients and found no significant differences in the frequencies of individual alleles. However, the CC genotype was significantly more prevalent in the CL/P group than in the healthy control group. Therefore, assessing the polymorphism in the promoter region of this gene can shed light on the etiopathogenesis of CL/P.

 The rs3918242 polymorphism (C/T) is functional in the promoter region of the MMP9 gene, which directly impacts nuclear protein binding and transcriptional activity. The T allele causes approximately a 1.5-fold increase in promoter activity and the subsequent upregulation of MMP9.^9^ Previous studies on palatal fusion have shown upregulation of MMP9 in the late stages of secondary palate formation and a subsequent down-regulation after fusion.^8^

 Furthermore, the present study examined the association of the rs3918242 polymorphism in the MMP9 gene with nonsyndromic CL/P in the Iranian population. The present results indicated a significant association, with a higher prevalence of the defective T allele in patients compared with healthy controls. Moreover, the TT genotype was more prevalent in the case group. These findings are consistent with the approximately 1.5-fold increase in promoter activity, potential MMP9 upregulation, and potentially increased risk of nonsyndromic CL/P. The findings of this study are contrary to the results of a study by Letra et al^30^ in Brazil. According to their results, the CC genotype was significantly more than CT in nonsyndromic CL/P patients, and none of the groups showed the TT genotype. Furthermore, Kumari et al^31^ in India found no significant association between nonsyndromic CL/P and MMP9 gene polymorphism.

 Nevertheless, it is noteworthy that the observed differences in the results of different studies may be due to factors such as sample size, geographical and environmental variations, and the multifactorial nature of these genes in CL/P development.

 Collectively, a comprehensive understanding of the influential factors in the occurrence of CL/P is crucial for developing more precise prevention and treatment strategies. The present study highlighted the significance of ongoing investigations into other genes and the necessity for a larger sample size and comprehensive analyses of diverse populations to reach more definite conclusions. With a deeper comprehension of the function of MMPs and their suppressors in the development of CL/P, potential therapeutic approaches and preventive strategies may be developed to address this congenital disorder.

## Conclusion

 This study found no significant association between MMP2 (rs243866) gene polymorphism and nonsyndromic CL/P in an Iranian population. However, a significant association was observed between MMP9 (rs3918242) gene polymorphism and nonsyndromic CL/P in the same population. Variations in the results of different studies could be attributed to differences in sample size and geographical and environmental factors, considering the multifactorial nature of these genes in the development of CL/P. Further research on larger and diverse populations is warranted to validate and elucidate the genetic contributions to nonsyndromic CL/P in various ethnic groups.

## Acknowledgments

 The authors would like to thank all the staff at the Genetic Research Center, University of Social Welfare and Rehabilitation Sciences, for cooperating in this study.

## Competing Interests

 The authors declare no conflicts of interest.

## Ethical Approval

 This study was approved by Research Ethics Committee of Research Institute of Dental Sciences-Shahid Beheshti University of Medical Sciences. (Ethics approval number: IR.SBMU.DRC.REC.1401.061).

## References

[1] Soghani B, Ebadifar A, Khorram Khorshid HR, Kamali K, Hamedi R, Aghakhani Moghadam F (2017). The study of association between reduced folate carrier 1 (RFC1) polymorphism and non-syndromic cleft lip/palate in Iranian population. Bioimpacts.

[2] Taher AA (1992). Cleft lip and palate in Tehran. Cleft Palate Craniofac J.

[3] Ebadifar A, Hamedi R, Khorram Khorshid HR, Saliminejad K, Kamali K, Aghakhani Moghadam F (2015). Association of transforming growth factor alpha polymorphisms with nonsyndromic cleft lip and palate in Iranian population. Avicenna J Med Biotechnol.

[4] Peng HH, Chang NC, Chen KT, Lu JJ, Chang PY, Chang SC (2016). Nonsynonymous variants in MYH9 and ABCA4 are the most frequent risk loci associated with nonsyndromic orofacial cleft in Taiwanese population. BMC Med Genet.

[5] Aylward A, Cai Y, Lee A, Blue E, Rabinowitz D, Haddad J Jr (2016). Using whole exome sequencing to identify candidate genes with rare variants in nonsyndromic cleft lip and palate. Genet Epidemiol.

[6] Iamaroon A, Wallon UM, Overall CM, Diewert VM (1996). Expression of 72-kDa gelatinase (matrix metalloproteinase-2) in the developing mouse craniofacial complex. Arch Oral Biol.

[7] Morris-Wiman J, Du Y, Brinkley L (1999). Occurrence and temporal variation in matrix metalloproteinases and their inhibitors during murine secondary palatal morphogenesis. J Craniofac Genet Dev Biol.

[8] Blavier L, Lazaryev A, Groffen J, Heisterkamp N, DeClerck YA, Kaartinen V (2001). TGF-beta3-induced palatogenesis requires matrix metalloproteinases. Mol Biol Cell.

[9] Zhang B, Henney A, Eriksson P, Hamsten A, Watkins H, Ye S (1999). Genetic variation at the matrix metalloproteinase-9 locus on chromosome 20q122-131. Hum Genet.

[10] Menezes-Silva R, Khaliq S, Deeley K, Letra A, Vieira AR (2012). Genetic susceptibility to periapical disease: conditional contribution of MMP2 and MMP3 genes to the development of periapical lesions and healing response. J Endod.

[11] Price SJ, Greaves DR, Watkins H (2001). Identification of novel, functional genetic variants in the human matrix metalloproteinase-2 gene: role of Sp1 in allele-specific transcriptional regulation. J Biol Chem.

[12] Yang J, Fan XH, Guan YQ, Li Y, Sun W, Yang XZ (2010). MMP-2 gene polymorphisms in type 2 diabetes mellitus diabetic retinopathy. Int J Ophthalmol.

[13] Pérez-Hernández N, Vargas-Alarcón G, Martínez-Rodríguez N, Martínez-Ríos MA, Peña-Duque MA, de la Peña-Díaz A (2012). The matrix metalloproteinase 2-1575 gene polymorphism is associated with the risk of developing myocardial infarction in Mexican patients. J Atheroscler Thromb.

[14] Cavalcante BGN, Lacerda RHW, Assis IO, Bezamat M, Modesto A, Vieira AR (2021). Talon cusp associates with MMP2 in a cohort of individuals born with oral clefts. Cleft Palate Craniofac J.

[15] Nasiri H, Forouzandeh M, Rasaee MJ, Rahbarizadeh F (2005). Modified salting-out method: high-yield, high-quality genomic DNA extraction from whole blood using laundry detergent. J Clin Lab Anal.

[16] Ghorbani F, Javadirad SM, Amirmahani F, Fatehi Z, Tavassoli M (2021). Associations of BCL2 CA-repeat polymorphism and breast cancer susceptibility in Isfahan province of Iran. Biochem Genet.

[17] Esmaeli Chamgordani L, Ebrahimi N, Amirmahani F, Vallian S (2020). CG/CA genotypes represent novel markers in the NPHS2 gene region associated with nephrotic syndrome. J Genet.

[18] Vahhab N, Ebrahimi N, Amirmahani F, Vallian S (2020). Analysis of polymorphic markers located in the HEXA gene region associated with Tay-Sachs disease. Meta Gene.

[19] Jamalvandi M, Motovali-Bashi M, Amirmahani F, Darvishi P, Jamshidi Goharrizi K (2018). Association of T/A polymorphism in miR-1302 binding site in CGA gene with male infertility in Isfahan population. Mol Biol Rep.

[20] Fatehi Z, Amirmahani F, Tavassoli M (2019). Association study of TAAAA polymorphism in the first intron of p53 gene with risk of colorectal cancer in Iranian population. Egypt J Med Hum Genet.

[21] Motovali-Bashi M, Amirmahani F, Ghatre Samani Z. Association between miR-152/148a polymorphisms and age of onset and progression of breast cancer in Isfahan population. Research in Medicine 2017;40(4):187-91. [Persian].

[22] Smane-Filipova L, Pilmane M, Akota I (2016). MMPs and TIMPs expression in facial tissue of children with cleft lip and palate. Biomed Pap Med Fac Univ Palacky Olomouc Czech Repub.

[23] Stetler-Stevenson WG (2008). The tumor microenvironment: regulation by MMP-independent effects of tissue inhibitor of metalloproteinases-2. Cancer Metastasis Rev.

[24] Letra A, Silva RA, Menezes R, Astolfi CM, Shinohara A, de Souza AP (2007). MMP gene polymorphisms as contributors for cleft lip/palate: association with MMP3 but not MMP1. Arch Oral Biol.

[25] Smane L, Pilmane M, Akota I (2013). Apoptosis and MMP-2, TIMP-2 expression in cleft lip and palate. Stomatologija.

[26] Brown NL, Yarram SJ, Mansell JP, Sandy JR (2002). Matrix metalloproteinases have a role in palatogenesis. J Dent Res.

[27] Werb Z, Chin JR (1998). Extracellular matrix remodeling during morphogenesis. Ann N Y Acad Sci.

[28] Machado RA, de Oliveira LQR, Ayroza Rangel ALC, de Almeida Reis SR, Scariot R, Martelli DRB (2022). Brazilian multiethnic association study of genetic variant interactions among FOS, CASP8, MMP2 and CRISPLD2 in the risk of nonsyndromic cleft lip with or without cleft palate. Dent J (Basel).

[29] Zalewska-Ziob M, Adamek B, Kasperczyk J, Łyko D, Płachetka A, Rokicki M (2014). Cleft lip and/or palate genetic conditioning–is MMP2 gene polymorphism important for this defect development?. Pediatr Med Rodz.

[30] Letra A, da Silva RA, Menezes R, de Souza AP, de Almeida AL, Sogayar MC (2007). Studies with MMP9 gene promoter polymorphism and nonsyndromic cleft lip and palate. Am J Med Genet A.

[31] Kumari P, Singh SK, Raman R (2019). TGFβ3, MSX1, and MMP3 as candidates for NSCL ± P in an Indian population. Cleft Palate Craniofac J.

